# Specific Antibody Deficiency: Controversies in Diagnosis and Management

**DOI:** 10.3389/fimmu.2017.00586

**Published:** 2017-05-22

**Authors:** Elena Perez, Francisco A. Bonilla, Jordan S. Orange, Mark Ballow

**Affiliations:** ^1^Allergy Associates of the Palm Beaches, North Palm Beach, FL, USA; ^2^Boston Children’s Hospital, Boston, MA, USA; ^3^Texas Children’s Hospital, Baylor College of Medicine, Houston, TX, USA; ^4^Division of Allergy and Immunology, Department of Pediatrics, University of South Florida, Saint Petersburg, FL, USA

**Keywords:** specific antibody deficiency, antibody deficiency, treatment, diagnosis, immunoglobulin replacement therapy, pneumococcal vaccines, primary immunodeficiency

## Abstract

Specific antibody deficiency (SAD) is a primary immunodeficiency disease characterized by normal immunoglobulins (Igs), IgA, IgM, total IgG, and IgG subclass levels, but with recurrent infection and diminished antibody responses to polysaccharide antigens following vaccination. There is a lack of consensus regarding the diagnosis and treatment of SAD, and its clinical significance is not well understood. Here, we discuss current evidence and challenges regarding the diagnosis and treatment of SAD. SAD is normally diagnosed by determining protective titers in response to the 23-valent pneumococcal polysaccharide vaccine. However, the definition of an adequate response to immunization remains controversial, including the magnitude of response and number of pneumococcal serotypes needed to determine a normal response. Confounding these issues, anti-polysaccharide antibody responses are age- and probably serotype dependent. Therapeutic strategies and options for patients with SAD are often based on clinical experience due to the lack of focused studies and absence of a robust case definition. The mainstay of therapy for patients with SAD is antibiotic prophylaxis. However, there is no consensus regarding the frequency and severity of infections warranting antibiotic prophylaxis and no standardized regimens and no studies of efficacy. Published expert guidelines and opinions have recommended IgG therapy, which are supported by observations from retrospective studies, although definitive data are lacking. In summary, there is currently a lack of evidence regarding the efficacy of therapeutic strategies for patients with SAD. We believe that it is best to approach each patient as an individual and progress through diagnostic and therapeutic interventions together with existing practice guidelines.

## Background

Specific antibody deficiency (SAD) is a primary immunodeficiency disease (PIDD) characterized by normal immunoglobulins (Igs), IgA, IgM, total IgG, and IgG subclass levels, but with recurrent infection and diminished antibody responses to polysaccharide antigens following vaccination (1–3). As with other forms of antibody deficiencies, it is most commonly associated with recurrent respiratory bacterial infections.

Specific antibody deficiency was first reported in both adult and pediatric patients in the 1980s (4–7). It was initially described as IgG_2_ subclass deficiency with an inability to generate measurable titers of antibody response to pneumococcal polysaccharides and is currently also termed partial antibody deficiency or impaired polysaccharide responsiveness (ICD-10 code D80.6) (8–10). There is some confusion concerning the relationship between SAD and IgG subclass levels and the controversial diagnosis of IgG subclass deficiency. More recently, these diagnoses are considered distinct, and the designation of SAD is reserved for impaired polysaccharide vaccine responsiveness with completely normal Ig isotype levels (11, 12). SAD mimics the deficient immune response often seen in healthy young children and infants who are unable to mount a robust response to pure unconjugated polysaccharide antigens such as *Streptococcus pneumoniae* polysaccharide and *Haemophilus influenzae* type b capsular polysaccharide. A healthy immune response normally develops by 2 years of age but may take longer in some children (2, 12, 13). As a result, the diagnosis should not be conferred until after 2 years of age (11, 12). It should be noted however, that some children under the age of 2 years (as young as 1 year) are able to mount robust responses to polysaccharide vaccines (14).

The incidence of SAD in the general population is unclear (2). SAD has been estimated to be the eighth most commonly identified PIDD globally (15); however, data regarding prevalence should be considered cautiously as they are based on reports from different centers based on different definitions of PIDD, and SAD may not be reported in all regions. In addition, the prevalence differs between referral populations and is dependent upon age and the serological definition of polysaccharide unresponsiveness in SAD, which has changed over the years (1). In three studies evaluating children for recurrent infection (*n* = 100, 45, and 100, respectively), SAD was found to occur in 6–14% of individuals (11, 16, 17). However, in a chart review of 91 children referred for immunologic evaluation of recurrent infections, 23.1% had been diagnosed with SAD (18). In addition, in 1 retrospective study of 129 adults with chronic rhinosinusitis, 11.6% were diagnosed with SAD (19), whereas in two other similar retrospective studies also of adults with chronic rhinosinusitis, 23% (*n* = 239) and 24% (*n* = 595) were diagnosed with SAD (20, 21). The prevalence of SAD in adults with recurrent pneumonia has been reported to be approximately 8% (22).

The origin and underlying molecular defects of SAD are not known (2), but decreased numbers of switched memory B-cells, which may play a key role in the protection against infection with polysaccharide-encapsulated bacteria, have been reported in patients with SAD (23). Patients are highly susceptible to severe respiratory tract infections with encapsulated bacteria, and SAD is one of the most commonly identified immune disorders in patients presenting with recurrent sinopulmonary infections (2, 20).

There is a lack of consensus regarding the diagnosis and treatment of SAD, and its clinical significance is not well understood. Here, we will discuss current evidence and challenges regarding the diagnosis and treatment of SAD.

## Diagnosis of SAD

The infections encountered in SAD are similar to those of other antibody deficiencies; patients typically present with recurrent upper and lower respiratory tract infections, otitis media, and sinusitis (1, 24). Asthma and rhinitis are also commonly reported in children with SAD (1), and the SAD phenotype may be found in other well-established PIDDs such as Wiskott–Aldrich syndrome, partial DiGeorge syndrome (24), and NF-κB essential modulator mutations (25). The phenotype of SAD can also be similar to that of common variable immunodeficiency (CVID). Variations exist in the diagnostic criteria for SAD versus CVID between the European Society for Immunodeficiency (ESID) criteria and US practice parameters, and also the definition of CVID from the International Consensus Document for CVID (Table 1) (3, 12, 26). Despite this, they are all in agreement that patients with CVID exhibit low IgG and usually IgA levels, and potentially low IgM levels, whereas patients with SAD exhibit normal IgG, IgA and IgM, and IgG subclass levels. SAD is generally less severe than CVID, and for some patients, the clinical features do not fit either definition precisely. SAD poses a diagnostic challenge as all Ig levels are normal (2), and it is frequently diagnosed only when all other causes are ruled out (27).

**Table 1:** **European Society for Immunodeficiency (ESID), US practice parameters, and International Consensus Document (ICON) criteria for the diagnosis of specific antibody deficiency (SAD) and common variable immunodeficiency (CVID) (3, 12, 26)**.

	SAD		CVID		
	ESID criteria (3)	US practice parameters (12)	ESID criteria (3)	US practice parameters (12)	ICON criteria (26)
Clinical presentation	Recurrent or severe bacterial infections	Recurrent respiratory tract infections	At least one of the following: increased susceptibility to infection autoimmune manifestations granulomatous disease unexplained polyclonal lymphoproliferation affected family member with antibody deficiency	Recurrent and chronic bacterial respiratory tract infections are the most frequent infectious complications Common pathogens include encapsulated or atypical bacteria Recurrent and/or persistent viral respiratory tract infections are also increased	Most patients will have at least 1 characteristic clinical manifestation (infection, autoimmunity, lymphoproliferation) Diagnosis may be conferred on asymptomatic individuals who fulfill other criteria listed below, especially in familial cases

Antibody levels	Normal IgG, IgA and IgM, and IgG subclass levels	Normal IgG, IgA and IgM, and IgG subclass levels	Marked decrease of IgG and IgA with or without low IgM levels	Low IgG and IgA levels with normal or low IgM levels	Serum IgG level must be below local/regional clinical laboratory norms Low IgA or IgM levels (low IgA preferred)

Response to vaccines	Profound alteration of the antibody responses to polysaccharide vaccine	Impaired response to pneumococcal capsular polysaccharide	At least one of the following: poor antibody response to vaccines (and/or absent isohemagglutinins) low switched memory B-cells	Impaired vaccine response	Impaired vaccine response

B-cells	Not considered	Normal B-cell levels	Possibly low switched memory B-cells (see criteria above)	Normal or low B-cell levels	Not considered

T-cells	Exclusion of T-cell defect	Not considered	No evidence of profound T-cell deficiency	Not considered	In patients with IgG >100 mg/dL, demonstrable impairment of response to T-cell antigens

Other diagnostic criteria	None	Patients older than 2 years	Secondary causes of hypogammaglobulinemia have been excluded Diagnosis is established after the 4th year of life (but symptoms may be present before)	Consider possible transient hypogammaglobulinemia Patients older than 4 years No genetic lesions or other causes of primary or secondary antibody deficiency	Other causes of hypogammaglobulinemia must be excluded

In patients with SAD, the prevalence of atopy is increased; in a study of 74 children with recurrent infection, allergic rhinitis was significantly associated with the presence of SAD (relative risk: 3.77; *p* = 0.04) (1). Allergic rhinosinusitis could contribute to susceptibility to infection in these patients. Specific IgE responses to environmental allergens should be sought by percutaneous testing and/or blood measurements. Diagnosis of SAD may be confounded in patients with allergy comorbidities, where aggressive treatment of allergies can diminish infection rates.

### Response to Pneumococcal Vaccines

**Figure 1:**
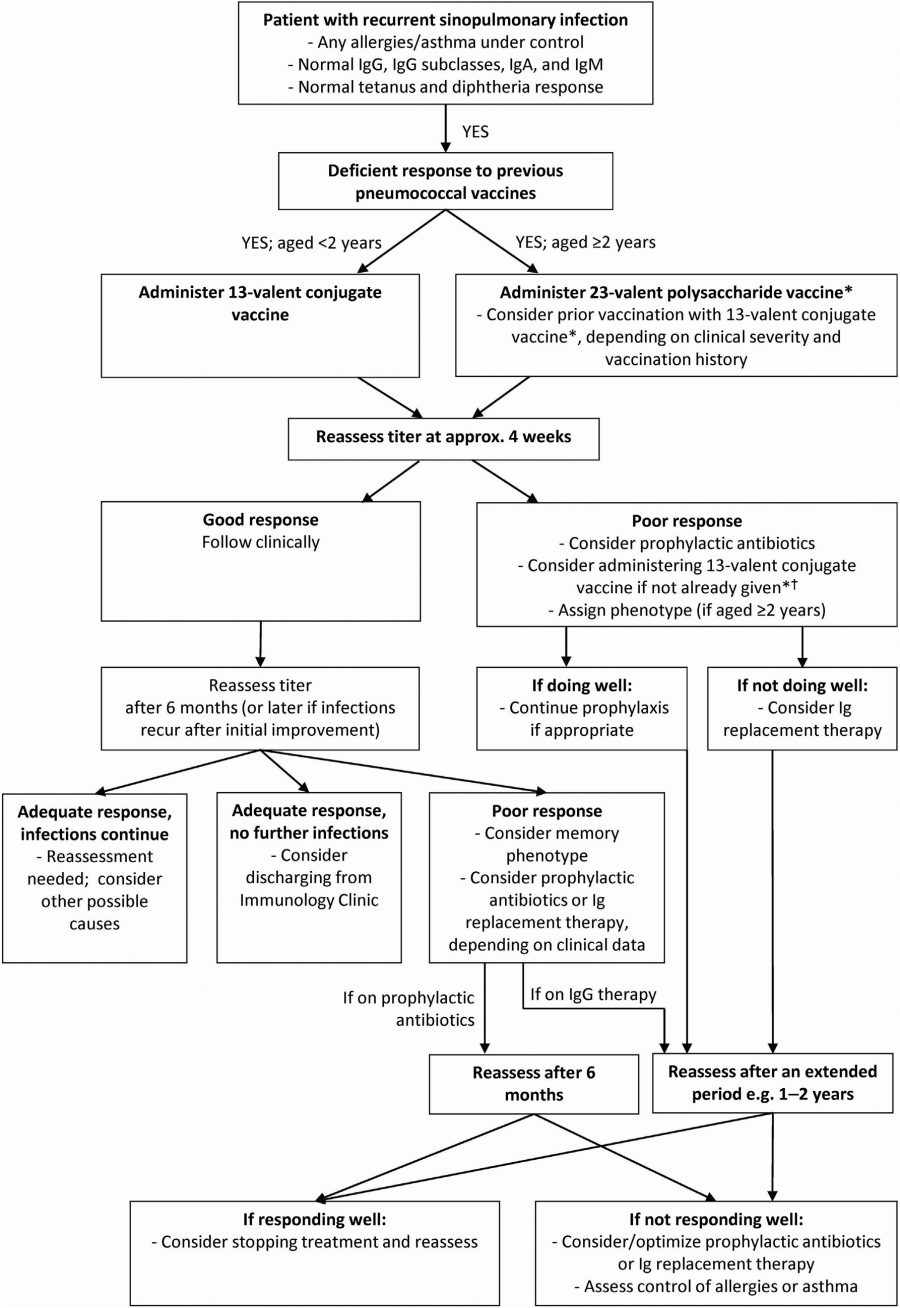
**Diagnosis and treatment algorithm for specific antibody deficiency (SAD)**. *The Centers for Disease Control and Prevention recommend that when both 23-valent pneumococcal polysaccharide vaccine (PPSV23) and 13-valent pneumococcal conjugate vaccine (PCV13) are indicated, PCV13 should be given before PPSV23 whenever possible. In adults PPSV23 should be given ≥8 weeks after previous doses of PCV13 and PCV13 should be given ≥1 year after the most recent dose of PPSV23. In patients 19–64 years of age PPSV23 may be revaccinated ≥5 years after last vaccination. In patients aged ≥65 years PPSV23 may be revaccinated once if ≥5 years after vaccination at<65 years of age. Additional doses of PCV13 should not be administered in patients ≥65 years. For further details of vaccination schedules, please refer to reference (28). ^†^Normal responses to PCV13 do not preclude diagnosis of SAD (12).

Specific antibody deficiency is characterized by an abnormal IgG antibody response to a pneumococcal vaccine, which was developed to protect against the Gram-positive cocci *S. pneumoniae* (12). The Centers for Disease Control and Prevention recommend 13-valent pneumococcal conjugate vaccine (PCV13) and 23-valent pneumococcal polysaccharide vaccine (PPSV23) for adults, and a series of PCV13 vaccinations for children under 2 years of age (28, 29). Vaccine indications and timings depend on age, previous vaccinations, and the presence of high-risk conditions including congenital immunodeficiency [see Figure 1 and Ref. (28) for further information].

Response to pneumococcal vaccines is usually determined by assessing levels of IgG specific for serotypes included in the vaccine by multiplex bead immunoassay or enzyme-linked immunosorbent assay (30–32). Radioimmunoassay has also been used in the past, although this method does not differentiate between antibody responses by different Ig isotypes (i.e., those other than IgG) (33). To date, the diagnosis of SAD has been hindered by a lack of controlled clinical studies and the absence of a standardized definition of an insufficient pneumococcal polysaccharide antibody response. Furthermore, and as alluded to above, the criteria for diagnosis as established by consensus groups have changed over the years.

### Diagnostic Thresholds and Controversies in Response to Polysaccharide Vaccines in the Diagnosis of SAD

Specific antibody deficiency is normally diagnosed by determining the ability to generate protective titers in response to pneumococcal vaccines (12); however, it is important to note that the definition of a protective titer is not uniform and may vary depending on the nature of the vaccine (12, 34, 35). A serotype-specific level of 1.3 μg/mL has been considered protective with respect to invasive disease following polysaccharide immunization (35, 36), and other studies have shown that levels of 0.35 µg/mL were deemed to provide protection against invasive pneumococcal infections following immunization with a conjugate pneumococcal vaccine (37). However, these studies are based on small cohorts and protective levels in response to pneumococcal vaccination and should be interpreted with caution (38). Furthermore, the level of specific antibody necessary to provide protection against infection in spaces such as the sinuses and middle ear has not been established.

An adequate response to immunization was previously defined as at least a fourfold increase in antibody levels over baseline for a given serotype (34, 39). However, the fourfold response criteria is no longer preferred as there is evidence to suggest that subjects with high baseline titers may not develop such an increase following vaccination (40). In a meta-analysis of antipneumococcal antibody responses in healthy individuals, the ratios of pre- to post-vaccination titers varied widely and depended on the particular serotype and the baseline level of antibody (36). One current recommendation is to accept a twofold response if the baseline level is ≥1.3 μg/mL (35), although the authors recommend that these data should be interpreted along with clinical correlation.

Controversy also exists over the precise number of pneumococcal serotypes needed to determine a normal response. This problem is compounded by the different pneumococcal vaccines that have been used historically. PPSV23 protects against 23 capsular serotypes (Table 2) and is a pure polysaccharide vaccine, meaning that it induces a T-cell-independent response by stimulating B-cells in the absence of T-helper cells (29). For this reason, polysaccharide vaccines are not reliably immunogenic in children under 2 years of age; thus, pneumococcal conjugate vaccines were developed, which generate a T-cell-dependent antibody response and are effective in children under 2 years (29). Earlier conjugate preparations contain 7 serotypes, while newer ones contain 13 (41). The availability of different vaccines with different antigens makes it difficult to standardize responses; for example, a retrospective report showed that PCV non-responders, including patients with SAD, may have good clinical and serological responses to PPSV23 (42).

**Table 2:** **Serotypes contained in pneumococcal vaccines, with permission from Ref. (41)**.

Serotypes	Vaccines		
	Heptavalent conjugate vaccine	13-valent conjugate vaccine	23-valent pneumococcal polysaccharide vaccine
1	–	✓	✓
2	–	–	✓
3	–	✓	✓
4	✓	✓	✓
5	–	✓	✓
6A	–	✓	–
6B	✓	✓	✓
7F	–	✓	✓
8	–	–	✓
9N	–	–	✓
9V	✓	✓	✓
10A	–	–	✓
11A	–	–	✓
12F	–	–	✓
14	✓	✓	✓
15B	–	–	✓
17F	–	–	✓
18C	✓	✓	✓
19A	–	✓	✓
19F	✓	✓	✓
20	–	–	✓
22F	–	–	✓
23F	✓	✓	✓
33F	–	–	✓

Furthermore, there has never been a study evaluating the correlation between degree of responsiveness and infection susceptibility. A working group report on diagnostic vaccination in PIDD recommend that a normal response to pneumococcal vaccines is a response to ≥50% of serotypes for patients under 6 years of age and a response to ≥70% of serotypes for patients over 6 years of age (35). In support of these specified thresholds, the meta-analysis of pneumococcal responses in healthy individuals showed that the majority of subjects could mount at least a twofold response to most serotypes (36). Recent attention has been focused upon other studies that report an adequate response as ≥1.3 μg/mL for >50% of serotypes (19, 40).

### Considerations for Severity of Deficiency in Response to Pneumococcal Polysaccharide Challenge

Although controversies exist regarding the definition of a protective titer, guidelines from a working group report were developed using the best evidence currently available to describe the diagnosis of mild, moderate, severe, and memory phenotypes of deficient response, based on response to PPSV23 (Table 3) (35). Patients with a mild phenotype have multiple serotypes to which they did not generate protective titers or were unable to increase titers twofold. Patients with a moderate phenotype produce protective titers to three or more serotypes but to <50% of serotypes for those under 6 years of age or <70% of serotypes for those over 6 years of age. A severe phenotype is described as producing protective titers against two or fewer serotypes, and those protective titers generated tend to be low. Patients with a memory phenotype of deficient responses initially mount an adequate response to vaccination but do not sustain the response beyond 6 months. It is important to note that pure polysaccharide vaccines invoke a T-cell-independent response and as such do not generate a long-lived memory B-cell response (although they can boost them if the patient has previously received a conjugate vaccine); the term “memory phenotype” refers to patients who lose an adequate response to PPSV23 more quickly than usual.

**Table 3:** Summary of deficient response phenotypes to the 23-valent pneumococcal polysaccharide vaccine (PPSV23), with permission from Ref. (35)*.

Phenotype^a^	Response to PPSV23, age >6 years	Response to PPSV23, age <6 years	Notes
Severe	≤2 protective titers (≥1.3 μg/mL)	≤2 protective titers (≥1.3 μg/mL)	Protective titers present are low
Moderate	<70% of serotypes are protective (≥1.3 μg/mL)	<50% of serotypes are protective (≥1.3 μg/mL)	Protective titers present to ≥3 serotypes
Mild	Failure to generate protective titers to multiple serotypes or failure of a twofold increase in 70% of serotypes	Failure to generate protective titers to multiple serotypes or failure of a twofold increase in 50% of serotypes	Twofold increases assume a pre-vaccination titer of <4.4–10.3 μg/mL, depending on the pneumococcal serotype
Memory	Loss of response within 6 months	Loss of response within 6 months	Adequate initial response to ≥50% of serotypes in children <6 years of age and ≥70% in those >6 years of age

*^a^All phenotypes assume a history of infection*.

*Reprinted from J Allergy Clin Immunol, 130, Orange J, Ballow M, Stiehm ER, et al. Use and interpretation of diagnostic vaccination in primary immunodeficiency: A working group report of the Basic and Clinical Immunology Interest Section of the American Academy of Allergy, Asthma & Immunology, S1-24, Copyright (2012), with permission from Elsevier.

For the diagnosis of SAD, titers against pneumococcal vaccine serotypes must be measured preimmunization and postimmunization (4 weeks after vaccination), and it is of greatest importance to consider whether the final antibody titer values are above protective limits. Fold increases are less relevant as patients with high preimmunization titers may not show a significant increase in antibody concentrations after vaccination (see above), so it may make sense not to include the serotype of a very high pre-vaccination titer in the post-vaccination analysis. Moreover, fold increases in patients with very low titers may be irrelevant (12). When considering the response to PPSV23, it is also imperative to take into account vaccination history. The practice parameter from the American Academy of Allergy, Asthma and Immunology (AAAAI), American College of Allergy, Asthma and Immunology (ACAAI), and Joint Council of Allergy, Asthma and Immunology (JCAAI) recommends that for patients who have previously received at least one dose of conjugate vaccine, normal antibody levels against serotypes in the conjugate vaccine do not exclude the diagnosis of SAD; thus, at least six serotypes should be tested that are present in PPSV23 only (12). Finally, without substantive and much needed natural history studies, it is also important to consider that SAD may be a transient issue in some patients, especially children, and may resolve over time (43).

### Variability in Response to Polysaccharide Vaccines with Age

Age has an important influence on the level of response to most pneumococcal polysaccharide antigens. The diagnosis of SAD should not be considered in patients less than 2 years of age where there is an overlap between the characteristic findings of SAD and those of healthy children. The responses to polysaccharide antigens, in particular, are less reliable in children under 2 years of age (13, 44). It has been demonstrated that some serotypes do not elicit a significant antibody response in small children, whereas others can elicit a high response; in one study, 78% of 1-year-old children had good antibody responses to >50% of serotypes in the pneumococcal polysaccharide vaccine (14). Another study has shown that children aged under 1 year were unable to mount an antibody response to serotype 14, but this serotype elicited the highest response in elderly individuals (45). Inversely, children aged 1 year mounted high antibody responses to serotype 3, whereas adult individuals were only able to mount weak responses (45). Thus, anti-polysaccharide antibody responses are age dependent and also likely to be serotype dependent. As further research is clearly needed in this area, patients cannot be reliably diagnosed based on polysaccharide vaccination response until they are over 2 years old and their immune system has matured sufficiently.

### Standardization of Diagnosis and Infections

It is extremely challenging to standardize the diagnostic approach for patients in whom SAD is suspected. The sentinel pathogen in patients with SAD is considered to be *S. pneumoniae* although its precise role in causing disease in these patients is not established. Typical infections include otitis media, sinusitis, bronchitis, and pneumonia (41); however, the objective documentation of upper and lower respiratory tract infections outside of pneumonia is poor. Sepsis, meningitis, and osteomyelitis may also occur, though less commonly. It is assumed in most cases, particularly in less clinically severe infections, that pneumococcus has a prominent role, although definitive microbiologic proof of its presence or causal relation to disease is rarely sought and often difficult to demonstrate. Culture data may be available for more invasive infections. The presence and pathogenicity of other common respiratory flora such as *H. influenzae, Moraxella catarrhalis*, etc. have not been studied, but are to some extent assumed along with the role for pneumococcus by extrapolation from studies of patients with antibody deficiencies who have similar presentations together with overt hypogammaglobulinemia (46). Contributions of viral agents to symptoms may also be assumed, but this also has not been formally studied. It should be noted that evidence from some small studies indicates that memory switched B-cell percentage is a good indicator of clinical complications associated with SAD (and also CVID) (23).

In any evaluation of patients for immunodeficiency, infections should be documented in as much detail as possible with appropriate culture and imaging data, and documentation of response to therapy. Other factors can come into play that may increase the frequency and severity of upper respiratory tract infections including smoking, daycare attendance, and atopic disease; these factors should be considered and treated or abated.

For patients who have best objective criteria for recurrent upper and/or lower respiratory tract infections, or those who have had a serious documented infection with pneumococcus or other bacterial pathogens (i.e., bacteremia) for which they have been vaccinated, consideration of the SAD diagnosis in those over 2 years of age is appropriate. Patients should have a standard screening immunological assessment consistent with the practice parameter to include quantitative Ig levels and titers (12). Assuming that other immunodeficiencies are not suspected based upon the laboratory tests obtained and the IgG level is normal, but antibody titers to pneumococcal polysaccharides are absent or low, then unconjugated pneumococcal vaccination should be administered (Figure 1). Titers should be measured at approximately 4 weeks thereafter. If the response is adequate, titers should be measured again 6 months after the vaccination in consideration of a possible “memory phenotype” of deficient vaccination response, or later if infections recur after initial improvement. If this assessment does not show substantive decreases in titer, the diagnosis of SAD should not be considered further. If the 4-week post-vaccination testing is abnormal, intervention can be considered based upon the severity of the hyporesponsiveness and clinical presentation.

## Therapeutic Intervention Options for Patients with SAD: Strategies and Unanswered Questions

Therapeutic strategies and options for patients with SAD are often based largely on clinical experience due to the lack of focused studies and absence of a robust case definition. However, a limited number of recommendations exist (Table 4). Most cases of SAD present with a relatively mild clinical phenotype, and the consensus is that these should be initially treated with antibiotic prophylaxis.

**Table 4:** **Therapeutic strategies for patients with specific antibody deficiency (SAD)**.

Recommendation by	Recommendation		
	Antibiotics	Immunoglobulin (Ig) replacement therapy	Vaccines
American Academy of Allergy, Asthma and Immunology American College of Allergy, Asthma and Immunology Joint Council of Allergy, Asthma and Immunology (12)	Treatment decisions should be based on the immunologic classification of mild, moderate, severe, and memory SAD		
	Patients with SAD might benefit from intensified use of antibiotics (grade of recommendation C)	In some cases patients with SAD might benefit from a period of IgG replacement therapy (grade of recommendation C) A determination can be made that IgG replacement is needed if they do not respond to other medical treatment; immunologic and clinical severity are the determining factors For patients who have responded to IgG replacement, selected patients who are deemed stable enough and are not likely to have a severe recurrence of symptoms can discontinue treatment after 1–2 years for a period of 4–6 months and then be re-evaluated. However, such treatment discontinuation must be deemed appropriate by the treating physician	Patients with SAD may benefit from additional immunization with conjugate pneumococcal vaccines (grade of recommendation C) If patients have not received the conjugate pneumococcal vaccine, immunization with the conjugate vaccine with the largest number of serotypes available is recommended in all patients with recurrent infections

Third National Immunoglobulin Database Report (UK) (51) AND Department of Health Recommendations (UK) (52)	Primary treatment	**Dose** Initiate at 0.4–0.6 g/kg/month; dose requirements may increase and should be based on clinical outcome **Criteria for administration** Approval by a clinical immunologist, AND Severe, persistent, opportunistic, or recurrent bacterial infections despite continuous oral antibiotic therapy for 3 months, AND Documented failure of serum antibody response to unconjugated pneumococcal or other poly saccharide vaccine challenge	[Not mentioned]

**Expert opinions**			

Wall et al. (24)	Antibiotic prophylaxis should be considered, especially in young patients who are likely to outgrow SAD	Indicated for patients with mild, moderate, or memory phenotypes who experience persistent infections despite appropriate management. In these patients, treatment should be discontinued after a period of 1–2 years and re-evaluated 4–6 months after discontinuation Patients with the severe phenotype or who have already developed permanent organ damage may be placed directly on Ig replacement and do not require re-evaluation	In patients with poor immunologic memory, re-immunization with 23-valent pneumococcal polysaccharide vaccine may re-establish protective antibody levels Most clinicians recommend waiting at least 1 year before re-immunization There is no indication to administer the vaccine again in patients who showed complete absence of response to an initial dose

Ocampo and Peters (40)	Yes	Yes	[Not mentioned]

Garcia-Lloret et al. (53)	Primary treatment	Ig replacement should only be for recurrent pyogenic infections poorly controlled with antibiotic therapy Children with SAD are started on intravenous Ig, the recommendation is to re-evaluate them after a year; if antibody responses improve and infections do not recur, therapy should be discontinued	[Not mentioned]

The practice parameter established by the ACAAI, AAAAI, and JCAAI recommends that patients with SAD may benefit from additional immunization with conjugate pneumococcal vaccines (12). If patients have not received the conjugate pneumococcal vaccine, immunization with the conjugate vaccine with the largest number of serotypes available is recommended in all patients with recurrent infections. Even in patients who had received the conjugate vaccine earlier in childhood, repeating this vaccination might lead to generation of antibody titers at a later point in life. The generation of titers from a conjugate vaccine, while not studied in a SAD population, should be measured and may provide therapeutic benefit. The mainstay of therapy for patients with SAD, however, is antibiotic prophylaxis (12). There are no standardized regimens and no studies of efficacy; all practice is based upon expert opinion.

As mentioned above, atopic disease is increased in patients with SAD (1). Allergies in these patients must be treated by standard interventions such as allergen avoidance, antihistamines, and topical steroids, along with treatments directed toward the defective antibody response (discussed below).

### Antibiotic Prophylaxis in Patients with SAD

There is little evidence to guide the use of antibiotic prophylaxis in patients with SAD, or indeed in patients with PIDDs, and current practice is not based on data from patients specifically diagnosed with SAD, but from immune competent patients with recurrent acute otitis media, chronic rhinosinusitis, cystic fibrosis, and bronchiectasis (24). For example, in 1 prospective double-blind study, 24 children were identified with bacterial respiratory infections that continued over a 4-month observation period. All of the 7 children treated with placebo continued to have bacterial respiratory infections, whereas 14 of 16 children treated with trimethoprim–sulfamethoxazole (TMP–SMX) became infection-free (*p* = 0.002) (47). Although the majority of experts use antibiotic prophylaxis in practice (48, 49), studies are needed to determine the optimal dose, duration, and choice of antibiotic. The topic of prophylactic antibiotics has recently been reviewed by Kuruvilla and de la Morena; however, SAD was not included in this discussion (50).

In practice, both antibiotic prophylaxis (and IgG) therapy are widely used to treat SAD. A survey of PIDD management of 405 allergists and immunologists in the United States was performed, and the findings were reported separately for general and specialized immunologists (based on patients with PIDD comprising more than 10% of their clinical practice) (49). The majority reported using antibiotic prophylaxis in patients with SAD, and there was no significant difference in the percentage of general and specialized immunologists using antibiotic prophylaxis. While less than 25% of general immunologists reported that this treatment was moderately or extremely useful, more than twice this number of specialized immunologists considered antibiotic prophylaxis useful and of value. We concur with this perspective and support the use of antibiotic prophylaxis as the first-line therapy where pneumococcal conjugate vaccination fails to provide protection. There is no consensus protocol regarding the frequency and severity of infections that should motivate clinicians to begin antibiotic prophylaxis; however, patients with two or more episodes of pneumonia in 1 year or with multiple (>4–5) episodes of otitis media or sinusitis could be considered for treatment. While the individual regimens applied to patients vary substantially, the AAAAI, ACAAI, and JCAAI practice parameter recommends regimens including azithromycin (500 mg weekly or 250 mg every other day in adults; 10 mg/kg weekly or 5 mg/kg every other day in children) and TMP–SMX (160 mg daily or twice daily in adults; 5 mg/kg daily or twice daily in children) (12). Some patients may require year-round prophylaxis; however, in patients who have seasonal variation in their susceptibility to infection, we have considered the seasonal application of antibiotic prophylaxis. We are unable to offer specific guidance as to duration of antibiotic prophylaxis, but patients should be evaluated every 6 months at least to assess interval history, infections, treatment and response, etc. Those who continue to have infections on antibiotic prophylaxis, or who cannot tolerate long-term antibiotics, should be considered as candidates for IgG supplementation.

### IgG Replacement Therapy in Patients with SAD

A number of published expert guidelines and opinions have recommended IgG therapy in patients with SAD (12, 24, 40, 51–53), as well as observations from retrospective studies (20, 54–56), although definitive data are lacking. The practice parameter from the AAAAI, ACAAI, and JCAAI recommends that some patients with SAD might benefit from a period of IgG replacement therapy, and that such a determination can be based on immunological and clinical severity, and unresponsiveness or adverse effects of other medical interventions (12). Other parameters outlined by the UK Department of Health recommend that IgG therapy should be given only if antibiotic therapy is ineffective and impaired antibody production is demonstrated (52, 53), which is consistent with opinions from other experts (24, 40, 53). Recommended doses of IgG for treatment of SAD are the same as those used to treat other PIDDs and are based on clinical outcome (52, 53); however, there are no evidence-based criteria to determine the optimal duration of IgG replacement therapy. The AAAAI, ACAAI, and JCAAI practice parameter recommends that young patients who have stabilized after a period of IgG treatment, and are deemed at low risk for relapse, should discontinue IgG treatment for a period of 4–6 months to re-evaluate therapy (12). Our experience suggests an initial IgG treatment duration of 6–12 months, depending on clinical circumstance, in which a patient has not taken prophylactic antibiotics is a reasonable approach. Following this, a consideration to stop IgG therapy may be made after an extended period of time, for example, 1–2 years, if the patient has been without infection and required no or very few courses of antibiotics. Patients with a poor response to prophylactic antibiotics should be reassessed after 6 months. Also, in some (but not all) patients, IgA and IgM levels, total B-cell numbers, and proportions of memory B-cells may also normalize over time and be used as a possible indicator of improvement in humoral immune function. It should be considered that spontaneous resolution of humoral immunodeficiency in children is more common than in adults.

The effectiveness of IgG therapy in preventing infections in patients with SAD has been assessed in retrospective studies. In an observational study of 91 children with recurrent respiratory infections, 10 patients with SAD were identified who did not respond to pneumococcal vaccine. IgG therapy was initiated in two of these children who had inadequate responses to four of the seven serotypes tested. Prior to diagnosis, 1 patient had experienced 25 episodes of acute otitis media and the other patient had suffered from 5 episodes of pneumonia and 3 of acute otitis media. Both children remained healthy during and after IgG treatment (55). In a retrospective study of 75 patients, 30 received 400 mg/kg/month IgG therapy as prophylaxis against recurrent infections that had continued despite antibiotic therapy and treatment of concomitant allergic diseases (54). Patients with fewer antibody responses to pneumococcal vaccination were more likely to require IgG therapy (*p* < 0.01), and the number of infections was significantly reduced following IgG therapy (*p* < 0.001). In another retrospective analysis, 20 patients with difficult-to-treat asthma were subsequently diagnosed with SAD. A dose of 400–600 mg/kg IgG administered intravenously every 3–4 weeks reduced morbidity, hospitalizations, and respiratory infections (56). In another retrospective chart review of 239 adults with chronic rhinosinusitis, 56 (23%) were diagnosed with SAD (20). Of these patients, 10 were treated with IgG replacement therapy. All 10 patients had fewer infections following IgG replacement therapy, and 7 were deemed to have “improved greatly.” Further studies are needed to evaluate the efficacy of IgG therapy in patients with SAD and to optimize treatment strategies.

The majority of immunologists in the US-based PIDD management survey mentioned above also reported using IgG therapy to treat at least some patients with SAD, and there was no significant difference in IgG use between specialized and general immunologists. A similar survey was also performed with experts from the AAAAI and also ESID (48). Similar to the previous survey, the results showed that approximately half of all immunologists recommended IgG therapy for at least 5–50% of patients with SAD and there was no significant difference between the percentages of specialized and general immunologists, nor in the percentages of experts from the AAAAI and ESID, who prescribe IgG therapy. These results indicate that although there is a lack of guidance regarding IgG use in patients with SAD, use does not appear to vary significantly by region or depending on the specialty of the immunologist.

In summary, although there is a lack of uniform recommendations and clear guidance for the treatment of SAD, we believe that it is most advisable to consider IgG therapy in those who have some combination of the following features: (a) severe or very frequent recurrent infections; (b) poor response to pneumococcal polysaccharide vaccination; (c) inability to tolerate antibiotic prophylaxis due to multiple hypersensitivity, severe side effects or complications such as *Clostridium difficile* colitis, etc.; or (d) failure to respond to prophylactic antibiotics.

## Future Directions

There are many areas where further studies are required to advance our understanding of SAD to guide and optimize diagnosis and treatment (Table 5) (24, 35). The natural history of SAD remains poorly described, and an understanding of the immunobiology of the disease could be invaluable in informing treatment decisions. Also, the prevalence of SAD in different age groups is unclear and further data, especially with regard to incidence in patients with specific types of infection, would aid diagnosis. SAD has often been considered an issue that may resolve with time, especially in children (55), but in others, it may evolve into more severe forms of humoral immunodeficiency such as CVID. Some evidence indicates that memory switched B-cell percentage is a good indicator of clinical complications associated with SAD (23); however, it is not effective at classifying patients according to SAD or CVID diagnosis and long-term studies are needed to understand which patients may face permanent impairment.

**Table 5:** **Evidence needed in specific antibody deficiency (SAD) (24, 35)**.

Evidence gap	Studies required
The diagnosis of SAD is not standardized	Good quality clinical studies are needed to facilitate accurate and early identification of the deficiency and to clearly define antibody responses that are indicative of SAD
Specific evidence gaps regarding diagnosis based on response to pneumococcal vaccines Normal response to polysaccharide vaccines Specific cutoff values Effect of repeat vaccination on antibody response	
Responses resulting from different sequential administration of different vaccine formulations	

The prevalence of SAD is unknown, which may hinder diagnosis	Epidemiological studies are required, especially with regard to incidence in patients with specific types of infection

The natural history of SAD remains elusive	An improved understanding of the immunobiology of the disease could better inform treatment decisions
It is not known which patients will improve over time and which will have a permanent deficiency	

Unclear who will benefit from IgG replacement therapy	Studies are needed to determine which patients with SAD will benefit from IgG replacement therapy, and when it should be administered
The number of cessations of IgG therapy before lifetime IgG replacement therapy is undefined	

Long-term outcome of patients who improve over time is unknown	Long-term studies are required to determine the outcome for patients who improve over time

Current recommendations are based on expert opinion and there is a lack of unified guidelines	Randomized, controlled trials are needed to determine the benefit of IgG replacement therapy in patients with SAD Data from good quality clinical trials will help to form unified guidelines

Further standardization of the diagnosis of SAD would also be extremely valuable and would facilitate accurate and early identification of patients, allowing for more effective therapeutic decisions. Variation in results from different laboratories also creates challenges for diagnosis based on the measurement of vaccine responses (57). Further clinical studies are needed to clearly define antibody responses that are indicative of SAD. Studies have already been conducted to identify if responses to a small number of specific serotypes in multivalent vaccines may be used to diagnose SAD more accurately, and although results are encouraging, further evidence is needed (1).

There is currently a lack of evidence regarding the efficacy of therapeutic strategies for patients with SAD. Robust studies are needed to define which patients would benefit from therapy of any kind and exactly what the best role is for prophylactic antibiotic regimens and IgG replacement therapy. Results will soon be available from a recently completed trial investigating the use of IgG therapy in SAD patients (NCT00522821) (58). Hopefully, these types of studies will provide clarity as to which patients benefit most from the currently available therapeutic options. For now, it is best to approach each patient as an individual and progress through diagnostic and therapeutic interventions in concert with existing practice guidelines.

## Author Contributions

All authors were responsible for conceiving, drafting, and critically revising this work, were accountable for the accuracy and integrity of the work, and gave final approval for publishing.

## Conflict of Interest Statement

EP has consulted for CSL Behring, Baxalta/Shire, and Grifols; has received royalties from UptoDate; serves on the Basic and Clinical Immunology Interest Section of the AAAAI as Vice Chair, has chaired the PIDD committee of the AAAAI, and is on the council for the Clinical Immunology Society. FB has consulted for CSL Behring, Grifols, and Shire. JO has consulted for CSL Bering, Grifols, ADMA Biologics, and Baxalta. MB has no conflicts of interest to declare.

## Abbreviations

AAAAI, American Academy of Allergy, Asthma and Immunology; ACAAI, American College of Allergy, Asthma and Immunology; CVID, common variable immunodeficiency; ESID, European Society for Immunodeficiency; ICON, International Consensus Document for CVID; Ig, immunoglobulin; JCAAI, Joint Council of Allergy, Asthma and Immunology; PCV13, 13-valent pneumococcal conjugate vaccine; PIDD, primary immunodeficiency disease; PPSV23, 23-valent pneumococcal polysaccharide vaccine; SAD, specific antibody deficiency; TMP–SMX, trimethoprim–sulfamethoxazole.
