# Corrigendum: Specific Antibody Deficiency: Controversies in Diagnosis and Management

**DOI:** 10.3389/fimmu.2018.00450

**Published:** 2018-03-15

**Authors:** Elena Perez, Francisco A. Bonilla, Jordan S. Orange, Mark Ballow

**Affiliations:** ^1^Allergy Associates of the Palm Beaches, North Palm Beach, FL, United States; ^2^Boston Children’s Hospital, Boston, MA, United States; ^3^Texas Children’s Hospital, Baylor College of Medicine, Houston, TX, United States; ^4^Division of Allergy and Immunology, Department of Pediatrics, University of South Florida, Saint Petersburg, FL, United States

**Keywords:** specific antibody deficiency, antibody deficiency, treatment, diagnosis, immunoglobulin replacement therapy, pneumococcal vaccines, primary immunodeficiency

**Error in Table and Table Legend**

In the original article there were mistakes in Table 3 regarding the numbers of protective titers (> and < were used instead of ≥ and ≤), and units of protective titers were described in mg/mL instead of μg/mL. Specific antibody deficiency phenotypes are correctly described as in Table 3 below. In addition, the acknowledgment of the original source of the data in the footnote has been amended to include that the article was reprinted from *J Allergy Clin Immunol*, 130, Copyright (2012). The correct footnote appears below.

**Table 3:** Summary of deficient response phenotypes to the 23-valent pneumococcal polysaccharide vaccine (PPSV23), with permission from Ref. (35)*.

Phenotype^a^	Response to PPSV23, age >6 years	Response to PPSV23, age <6 years	Notes
Severe	≤2 protective titers (≥1.3 μg/mL)	≤2 protective titers (≥1.3 μg/mL)	Protective titers present are low
Moderate	<70% of serotypes are protective (≥1.3 μg/mL)	<50% of serotypes are protective (≥1.3 μg/mL)	Protective titers present to ≥3 serotypes
Mild	Failure to generate protective titers to multiple serotypes or failure of a twofold increase in 70% of serotypes	Failure to generate protective titers to multiple serotypes or failure of a twofold increase in 50% of serotypes	Twofold increases assume a pre-vaccination titer of <4.4–10.3 μg/mL, depending on the pneumococcal serotype
Memory	Loss of response within 6 months	Loss of response within 6 months	Adequate initial response to ≥50% of serotypes in children <6 years of age and ≥70% in those >6 years of age

*^a^All phenotypes assume a history of infection*.

*Reprinted from J Allergy Clin Immunol, 130, Orange J, Ballow M, Stiehm ER, et al. Use and interpretation of diagnostic vaccination in primary immunodeficiency: A working group report of the Basic and Clinical Immunology Interest Section of the American Academy of Allergy, Asthma & Immunology, S1-24, Copyright (2012), with permission from Elsevier.

**Text Corrections**

In the original article, there were errors in the text where the erroneous parameters from Table 3 were described. A serotype-specific protective level of 1.3 mg/mL in response to pneumococcal vaccination was discussed; the correct level is 1.3 µg/mL. This correction has been made to the section on Diagnostic Thresholds and Controversies in Response to Polysaccharide Vaccines in the Diagnosis of SAD, paragraph one. In the same paragraph, ‘of’ has been changed to ‘and’ in the third sentence:

‘Specific antibody deficiency (SAD) is normally diagnosed by determining the ability to generate protective titers in response to pneumococcal vaccines (12); however, it is important to note that the definition of a protective titer is not uniform and may vary depending on the nature of the vaccine (12, 34, 35). A serotype-specific level of 1.3 µg/mL has been considered protective with respect to invasive disease following polysaccharide immunization (35, 36), and other studies have shown that levels of 0.35 µg/mL were deemed to provide protection against invasive pneumococcal infections following immunization with a conjugate pneumococcal vaccine (37). However, these studies are based on small cohorts and protective levels in response to pneumococcal vaccination and should be interpreted with caution (38). Furthermore, the level of specific antibody necessary to provide protection against infection in spaces such as the sinuses and middle ear has not been established.’

Patients with a moderate SAD phenotype were described in the original article as those who ‘produce protective titers to more than three serotypes’; the correct number is ‘three or more serotypes’. These corrections have been made to the section on Considerations for Severity of Deficiency in Response to Pneumococcal Polysaccharide Challenge, paragraph one:

‘Although controversies exist regarding the definition of a protective titer, guidelines from a working group report were developed using the best evidence currently available to describe the diagnosis of mild, moderate, severe, and memory phenotypes of deficient response, based on response to PPSV23 (Table 3) (35). Patients with a mild phenotype have multiple serotypes to which they did not generate protective titers or were unable to increase titers twofold. Patients with a moderate phenotype produce protective titers to three or more serotypes but to <50% of serotypes for those under 6 years of age or <70% of serotypes for those over 6 years of age. A severe phenotype is described as producing protective titers against two or fewer serotypes, and those protective titers generated tend to be low. Patients with a memory phenotype of deficient responses initially mount an adequate response to vaccination but do not sustain the response beyond 6 months. It is important to note that pure polysaccharide vaccines invoke a T-cell-independent response and as such do not generate a long-lived memory B-cell response (although they can boost them if the patient has previously received a conjugate vaccine); the term “memory phenotype” refers to patients who lose an adequate response to PPSV23 more quickly than usual.’

The authors apologize for these errors and state that they do not change the scientific conclusions of the article in any way. The original article was updated.

## Conflict of Interest Statement

EP has consulted for CSL Behring, Baxalta/Shire, and Grifols and has received royalties from UptoDate. FB has consulted for CSL Behring, Grifols, and Shire. JO has consulted for CSL Behring, Grifols, ADMA Biologics, and Shire and lectured for Shire. MB has lectured and participated in advisory boards for CSL Behring, Shire, and Grifols and acted on Data Safety Monitoring Boards for Kedrion, CSL Behring, Green Cross, and Prometic.
